# Structural and electrical characterization of Hf_0.5_Zr_0.5_O_2_ thin films crystallized by rapid thermal annealing

**DOI:** 10.1186/s42649-026-00141-x

**Published:** 2026-06-16

**Authors:** Jucheol Park, Yeong Gyeong Park, Min-Ho Kang, Myung-Keun Lee, Moon Seop Hyun

**Affiliations:** 1 KENTECH Shared Research Facility, Korea Institute of Energy Technology, 21 Kentech-Gil, Naju, 58330 Republic of Korea; 2 Gumi Electronics & Information Technology Research Institute (GERI), Gumi, 39171 Republic of Korea; 3 National NanoFab Center (NNFC), Daejeon, 34141 Republic of Korea

**Keywords:** Ferroelectric Hf_0.5_Zr_0.5_O_2_, Piezoresponse force microscopy, Rapid thermal annealing, Grazing incidence X-ray diffraction, Transmission electron microscopy

## Abstract

The effects of rapid thermal annealing (RTA) temperature on the structural and electrical properties of 10 nm-thick Hf_0.5_Zr_0.5_O_2_ (HZO) thin films were investigated. Structural analyses revealed that the films remained largely amorphous at low annealing temperatures and crystallized above 600 °C, where the ferroelectric orthorhombic phase became dominant. At higher temperatures, the formation of the monoclinic phase was also observed. Electrical characterization demonstrated a strong dependence of ferroelectric behavior on annealing temperature. Films annealed near 600 °C exhibited the most stable ferroelectric domains, strongest polarization switching, and most distinct hysteretic behavior, whereas higher temperatures led to a gradual degradation of the electrical response. These results indicate that an annealing temperature around 600 °C provides the optimum condition for achieving enhanced ferroelectric performance in HZO thin films.

## Introduction

Hafnium–zirconium oxide (Hf_0.5_Zr_0.5_O_2_, HZO) has emerged as a leading ferroelectric material for CMOS‑integrated nonvolatile memory, logic, and neuromorphic hardware owing to its high permittivity, thermal stability, scalability to a few nanometers, and full process compatibility with advanced silicon platforms (Wilk et al. 2001 and Park et al. 2018). Building on the industrial adoption of fluorite‑structured HfO_2_/ZrO_2_ as high‑k gate dielectrics in MOSFETs and DRAM capacitor dielectrics, HZO uniquely exhibits robust room‑temperature ferroelectric switching at thicknesses where conventional perovskites struggle, positioning it as a practical pathway to dense, low‑power ferroelectric devices (Chernikova et al. 2016; Böscke et al. 2011; Müller et al. 2012; Polakowski and Muller 2015; Robertson et al. 2004).

The ferroelectric response in HZO originates from a metastable, non-centrosymmetric orthorhombic phase (commonly denoted Pca2_1_ in the standard setting; equivalent polar settings such as Pbc2_1_ are frequently used in thin‑film studies). This phase competes with the nonpolar tetragonal P4_2_/nmc and the thermodynamically stable monoclinic P2_1_/c phases (Huan et al. 2014; Materlik et al. 2015; Sang et al. 2015). Consequently, maximizing the fraction, preferred orientation, and stability of the orthorhombic phase —by increasing its volume fraction, aligning its polar axis favorably with respect to the electric field, and maintaining its metastable structure against phase transformation— is essential for achieving optimal ferroelectric device performance (Park et al. 2018; Mikolajick et al. 2021).

However, the phase stability of HZO is highly sensitive to processing conditions, including film thickness, Hf:Zr composition, oxygen vacancy concentration, electrode material (e.g., TiN), mechanical boundary conditions, and, most critically, the post-deposition thermal treatment (Chernikova et al. 2016). Among these factors, rapid thermal annealing (RTA) plays a decisive role in governing phase evolution. Insufficient annealing can leave the film amorphous or poorly crystallized, thereby suppressing polarization, whereas excessive annealing can relieve strain, alter defect distributions, and promote transformation to the monoclinic ground state, ultimately degrading ferroelectric properties. These effects become even more pronounced in ultrathin films (~ 10 nm), where interfacial energy and mechanical constraints can dominate over bulk thermodynamics (Müller, et al., 2011; Starschich et al. 2017; Yu et al. 2021).

To systematically enhance ferroelectricity in fluorite‑structured HfO_2_‑based systems, it is crucial to establish a clear understanding of how the polar orthorhombic phase forms at the microscopic level and how it governs the macroscopic electrical response. Despite significant progress, important gaps remain in constructing a consistent, multiscale structure–property relationship for ultrathin HZO films across a wide range of rapid thermal annealing (RTA) conditions.

First, the optimal RTA window for maximizing the orthorhombic phase fraction and stabilizing ferroelectric switching in ultrathin (~ 10 nm) films is not yet well defined and is often highly process-dependent. Systematic studies over a wide range of annealing temperatures are still limited. Second, phase identification based on diffraction techniques requires careful validation through real-space observations at the nanoscale, as factors such as preferred orientation, peak overlap, and phase coexistence can complicate interpretation.

Furthermore, discrepancies can arise between nanoscale electromechanical responses—such as PFM amplitude/phase contrast and local switching behavior—and macroscopic electrical characteristics, including C–V hysteresis. These differences are closely related to the evolution of defects, internal stress, and domain-wall pinning with annealing temperature. Therefore, a unified, multiscale approach that correlates structural, microscopic, and electrical measurements is essential for a comprehensive understanding of ferroelectric behavior in HZO films (Park et al. 2015 and Starschich et al. 2014).

In this work, we systematically investigate 10 nm-thick HZO films subjected to rapid thermal annealing (RTA) over a broad temperature range, with a focus on correlating structural evolution with electrical response. Grazing-incidence X-ray diffraction, cross-sectional TEM, and atomic-resolution HAADF-STEM with FFT analysis collectively reveal the onset of crystallization, microstructural characteristics, and the predominance of the orthorhombic (Pbc2_1_) phase in annealed HZO films.

To establish a comprehensive structure–property relationship, electrical characterization was performed using complementary techniques— electrostatic force microscopy (EFM) for probing bias-dependent surface potential, piezoresponse force microscopy (PFM) for imaging ferroelectric domains and switching behavior, and C–V measurements on TiN/HZO/TiN capacitors to evaluate macroscopic electrical properties.

## Materials and methods

### Fabrication of Hf_0.5_Zr_0.5_O_2_ thin film capacitor structures

10 nm-thick Hf_0.5_Zr_0.5_O_2_ (HZO) films were deposited by thermal atomic layer deposition (ALD) using a CN-1 (Korea) multi-chamber ALD system on Si substrates with a 100 nm-thick SiO_2_ interfacial layer. HfO_2_ and ZrO_2_ were alternately deposited at a substrate temperature of 250 °C using tetrakis (ethylmethylamido) hafnium (TEMAHf) and tetrakis (ethylmethylamido) zirconium (TEMAZr) as the hafnium and zirconium precursors, respectively, with ozone (O_3_) as the reactant and argon (Ar) as the purge gas. For HfO_2_, the ALD cycle consisted of a source dosing time of 0.7 s, source purge of 4.0 s, reactant dosing of 1.0 s, and reactant purge of 6.0 s. For ZrO_2_, the corresponding times were 1.5 s, 10.0 s, 1.0 s, and 10.0 s, respectively. Both HfO_2_ and ZrO_2_ exhibited a growth per cycle (GPC) of approximately 1.0 Å/cycle, and the HZO films were grown by alternating HfO_2_ and ZrO_2_ deposition in a 1:1 ratio with a total of 50 cycles.

For structural and electrical characterization, different sample configurations were prepared: HZO/SiO_2_/Si for TEM and XRD, HZO/TiN for EFM and PFM, and TiN/HZO/TiN for TEM and C-V analysis. The TiN electrodes were deposited by physical vapor deposition (PVD) using an AMAT (USA) 200 mm Endura-5500 sputtering system at a substrate temperature of 300 °C with a DC power of 6.5 kW, using a mixed Ar/N_2_ gas flow of 25/65 sccm at a process pressure of 3.7 mTorr. For C-V measurements, 200 nm-thick TiN was deposited as both top and bottom electrodes, and the top TiN electrode was patterned by wet etching using an NH₄OH:H₂O₂:H₂O solution with a concentration ratio of 1:2:5.

The HZO films were subsequently crystallized by post-metal rapid thermal annealing (RTA) in the temperature range of 400–1000 °C using a Metron (USA) 200 mm AG Heatpulse system. The annealing was performed with a ramp rate of 50 °C/sec and a dwell time of 30 s at each target temperature, followed by natural convection cooling. All processes were carried out under a nitrogen (N_2_) atmosphere at a flow rate of 5 SLPM, and no additional surface treatment was applied before or after the RTA process.

### Analysis methods

Grazing-incidence X-ray diffraction (GI-XRD) measurements were first performed to analyze the crystal structure of the HZO films after post-metal annealing. The measurements were carried out using a Rigaku SmartLab diffractometer with Cu Kα radiation (λ = 1.5406 Å). A fixed incidence angle (typically ~ 0.5°–1°) was employed to enhance surface sensitivity and minimize substrate contributions, and diffraction patterns were collected over a 2θ range of 20–50° with an appropriate step size and scan rate to resolve phase-related peaks.

Transmission electron microscopy (TEM) analysis was subsequently conducted on HZO thin films subjected to RTA at various temperatures to directly observe microstructural evolution. Cross-sectional TEM images were acquired at a scale of 10 nm to examine thickness uniformity, interface quality, and crystallization behavior as a function of annealing temperature. The crystalline phases were further characterized using conventional high-resolution transmission electron microscopy (HRTEM) and high-angle annular dark-field scanning transmission electron microscopy (HAADF-STEM) with a JEOL ARM-200F equipped with a cold field emission gun and a spherical aberration (Cs) corrector.

Electrostatic force microscopy (EFM) was employed to investigate the microscale and nanoscale electrical properties of the HZO surface in the absence of a top electrode, using an NX-10 AFM system. The measurements consisted of sequential writing and reading processes to evaluate the ferroelectric response. During the writing step, a PPP-EFM cantilever (resonant frequency ~ 70 kHz) was operated in contact mode to enable effective dipole switching through direct tip–sample interaction. A positive bias of + 3 V was first applied over a 3 μm × 3 μm area, followed by a negative bias of − 3 V applied to a smaller region of 1.5 μm × 1.5 μm, thereby inducing oppositely polarized domains.

Subsequently, the reading process was carried out in non-contact EFM mode using the same cantilever to ensure measurement consistency. The sample was maintained at 0 V (grounded) to avoid unintended polarization during imaging. A larger scan area of 5 μm × 5 μm was selected to capture both written and unwritten regions, allowing clear visualization of the resulting surface potential contrast associated with the ferroelectric domain configuration.

Piezoresponse force microscopy (PFM) measurements were performed using the same cantilever as in the EFM experiments to ensure consistency. During the writing process, the probe was operated in contact mode, and a DC bias of + 5 V was applied to the bottom electrode while scanning a 2 μm × 2 μm area, followed by − 5 V over a smaller 1.0 μm × 1.0 μm region to create oppositely polarized domains. For the reading process, PFM imaging was conducted over a 3 μm × 3 μm area with the sample grounded (0 V DC bias) while applying an AC modulation voltage of 1 V to detect the piezoresponse signal.

Capacitance–voltage (C–V) measurements were carried out using an Agilent E4980A. The DC bias was swept from − 3 V to + 3 V, while a small AC signal with a frequency of 1 kHz and an amplitude of 0.05 V (peak-to-peak) was superimposed to evaluate the dielectric and ferroelectric response of the capacitor structures.

## Results and discussion

### Structural characteristics of HZO thin films with various RTA temperatures

**Fig. 1:**
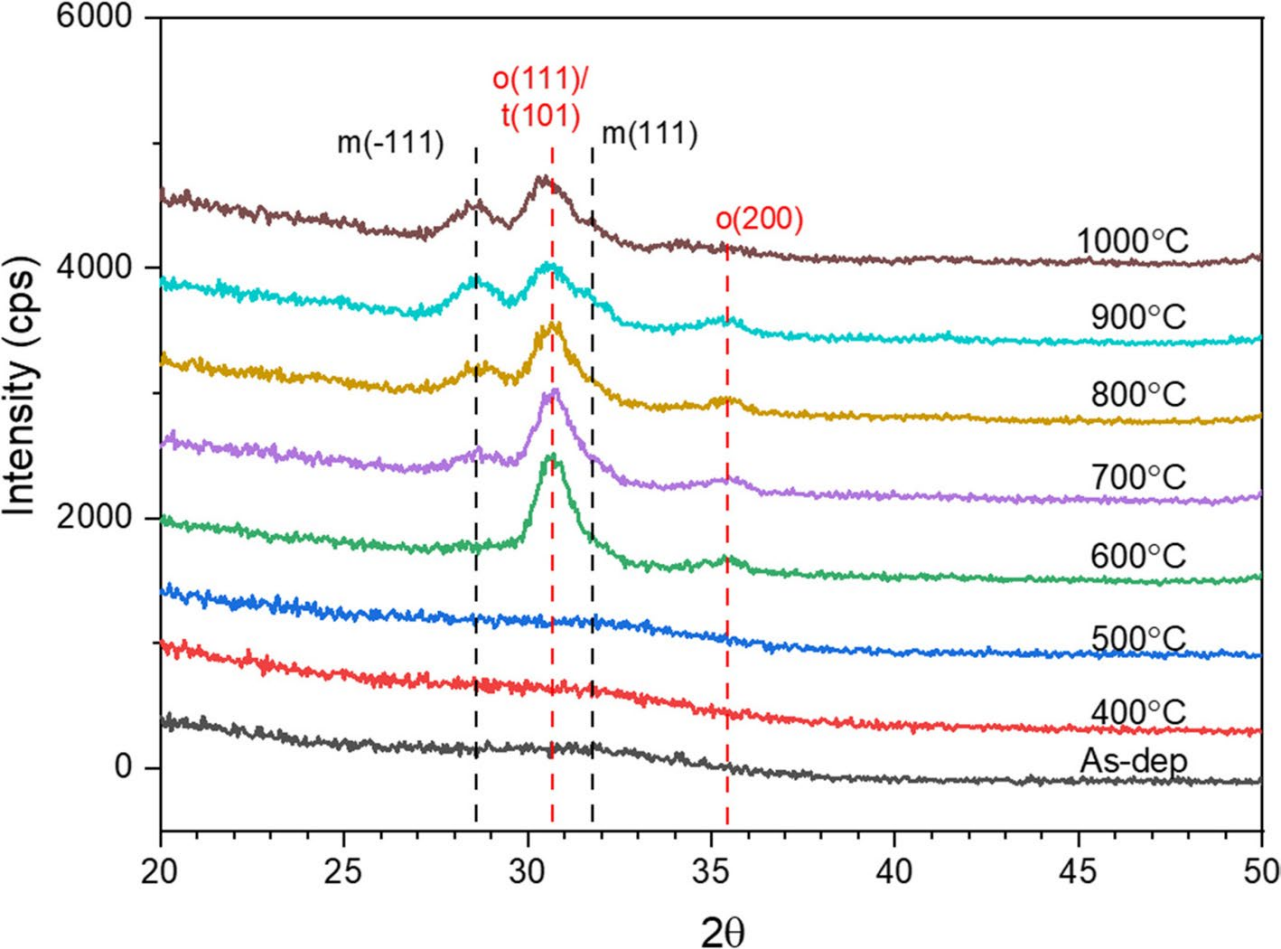
Grazing incidence X-ray diffraction (GI-XRD) patterns of Hf_0.5_Zr_0.5_O_2_ thin films as a function of post-deposition annealing temperature from the as-deposited state to 1000 °C. Vertical dashed lines denote characteristic diffraction peaks assigned to the monoclinic (black) and orthorhombic (red) phases: the m(−111) and m(111) peaks at ~ 28.5° and ~ 31.5° at higher temperatures, o-(111) and o-(200) at ~ 30.5°and ~ 35.5°

Grazing-incidence X-ray diffraction (GI-XRD) measurements were carried out on both as-deposited and RTA-annealed HZO films at various temperatures to investigate their crystalline properties. Figure 1 presents the GI-XRD patterns of the 10 nm-thick HZO films as a function of annealing temperature. The as-deposited sample showed a typical spectrum of an amorphous material with no special crystal peaks. No significant peaks were found in the samples at the 400 ℃, and the crystalline peaks began to be found in the 600 ℃ sample, with the number of peaks increasing with increasing RTA temperature.

Three prominent diffraction peaks are observed at 2θ ≈ 28.5º, 30.5º and 35.5º within the range of 25–40°. The peak at 2θ ≈ 28.5° is attributed to the (− 111) reflection of the monoclinic P2_1_/c phase. The dominant peak near 2θ ≈ 30.5° arises from overlapping contributions of the (011) reflection of the orthorhombic Pbc2_1_ phase and the (111) reflection of the tetragonal P4_2_/nmc phase (Hachemi et al. 2021). In addition, a weaker peak at 2θ ≈ 35.5° corresponds to the (200) reflection of the orthorhombic phase (Kim et al. 2022).

Analysis of the peak evolution with increasing RTA temperature (Fig. 1) reveals that the orthorhombic phase reaches its maximum intensity at 600 °C and subsequently decreases at higher temperatures. In contrast, the monoclinic phase emerges at around 700 °C and becomes more pronounced with further temperature increase. These results indicate that the optimal temperature for stabilizing the orthorhombic phase is around 600 °C.

**Fig. 2:**
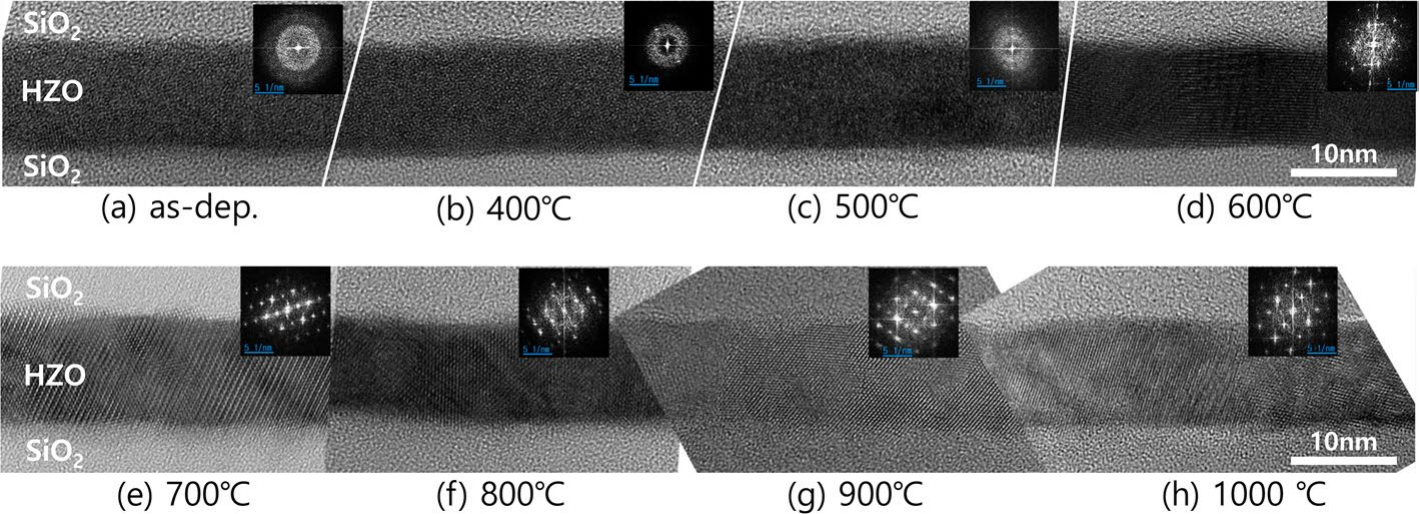
Cross‑sectional TEM images of SiO_2_/HZO(Hf_0.5_Zr_0.5_O_2)_/SiO_2_ stacks after rapid thermal annealing (RTA) at (**a**) as‑deposited, (**b**) 400 °C, (**c**) 500 °C, (**d**) 600 °C, (**e**) 700 °C, (**f**) 800 °C, (**g**) 900 °C, and (**h**) 1000 °C

Figure 2 shows cross-sectional TEM images of HZO/SiO_2_/Si structures for the as-deposited film and samples annealed at various RTA temperatures, providing complementary insight to the XRD phase analysis. The HZO layer maintains a uniform thickness of approximately 10 nm with relatively smooth surfaces and well-defined interfaces under all conditions. In the as-deposited state (Fig. 2a), the HZO layer exhibits a homogeneous, featureless contrast devoid of resolvable lattice fringes, and its FFT displays a diffuse, isotropic halo ring centered about the transmitted beam. At 400 °C and 500 °C, the bulk of the film remains predominantly amorphous; however, faint but discernible lattice fringes appear locally at the HZO/SiO_2_ interface, and the corresponding FFT patterns exhibit a few discrete spots superimposed on the residual diffuse halo. These reflections, combined with the lattice fringes seen only at the interface, shows that crystallization begins at the HZO/SiO_2_ interface rather than in the bulk. Because the interface is energetically favorable, small oriented nuclei form there first, ahead of crystallization in the bulk of the film. The onset of bulk crystallization becomes evident at 600 °C. The lattice fringes now extend from the interface into the interior of the film, and the FFT develops several many distinct spots, indicating that nanocrystalline grains are forming throughout the HZO film. After annealing at 700 °C and 800 °C, continuous and well-defined lattice fringes spread throughout the HZO layer, while the FFT patterns sharpen into clearly indexable diffraction spots. These features confirm the development of long-range crystallographic order and significant grain growth. At 900 °C and 1000 °C, the lattice images show coarse, faceted grains with distinct boundaries extending through the full film thickness, while the FFT patterns develop into sharp, single-crystal-like spot arrays typical of large, well-oriented crystallites. These observations are consistent with the XRD results, confirming that crystallization first appears as localized ordering at the interface at 400–500 °C, spreads into the bulk near 600 °C, and finally leads to extensive grain growth above 700 °C.

**Fig. 3:**
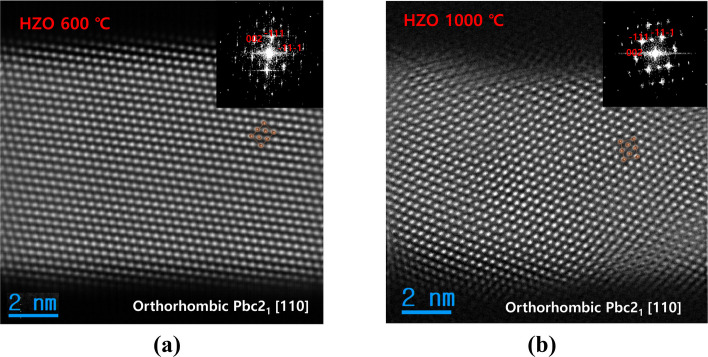
Atomic‑resolution HAADF‑STEM images of Hf_0.5_Zr_0.5_O_2_ (HZO) films after rapid thermal annealing, showing the orthorhombic Pbc2_1_ phase. **a** 600 °C, [110] zone; (**b**) 1000 °C, [110] zone. Insets display FFTs with representative indexed reflections consistent with Pbc2_1_ symmetry. The overlaid orange atomic schematic represents the projected two-dimensional unit cell of the orthorhombic Pbc2₁phase along the [110] direction

Figure 3 presents atomic-resolution HAADF-STEM images of Hf_0.5_Zr_0.5_O_2_ films annealed at 600 °C and 1000 °C, viewed along different zone axes, together with their corresponding FFT patterns (insets). In both cases, the lattice fringes and the discrete FFT spots can be indexed to the orthorhombic Pbc2₁phase. For direct comparison with the experimental atomic columns, the projected two-dimensional unit cell of the orthorhombic Pbc2₁phase along the [110] direction is overlaid as an orange schematic. These observations indicate that the ferroelectric orthorhombic phase is stabilized and remains predominant after annealing above 600 °C, in good agreement with the X-ray diffraction results.

### Electrical characteristics of HZO/TiN structures with various RTA temperatures

To evaluate the effect of RTA temperature on the electrical properties of HZO films, electrostatic force microscopy (EFM) and piezoresponse force microscopy (PFM) were employed to probe the electrostatic and electromechanical responses, respectively. In EFM, the quad signal is particularly sensitive to capacitance variations between the conductive tip and the sample surface, reflecting the local dielectric and polarization behavior.

**Fig. 4:**
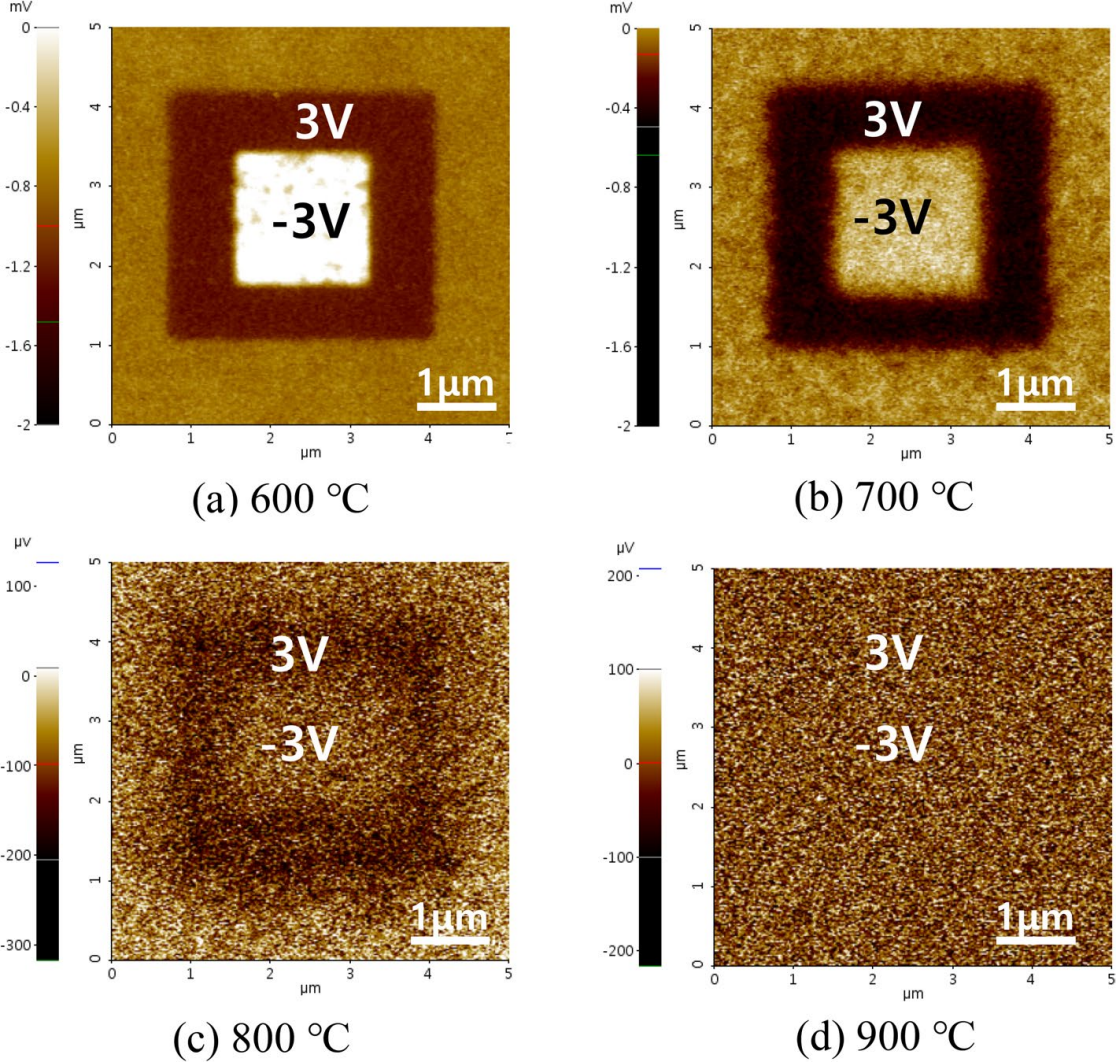
Electrostatic force microscopy (EFM) quad images of HZO thin films after rapid thermal annealing at different temperatures: (**a**) 600 °C, (**b**) 700 °C, (**c**) 800 °C, and (**d**) 900 °C

Figure 4 presents EFM quad images of samples annealed at 600 °C, 700 °C, 800 °C, and 900 °C, acquired over a 5 μm × 5 μm area under applied biases of ± 3 V. The sample annealed at 600 °C exhibits a pronounced voltage contrast between the positively and negatively biased regions, corresponding to a high surface potential of 1.515 mV. As the annealing temperature increases, the contrast progressively diminishes: the surface potential decreases to 0.535 mV at 700 °C and further to 0.029 mV at 800 °C. At 900 °C, the contrast becomes nearly indistinguishable, with a minimal surface potential of 0.004 mV. These results indicate that increasing the annealing temperature leads to a systematic reduction in the EFM voltage response, implying changes in the electrical properties of the HZO films, such as decreased ferroelectric polarization and a weakened dipole response at higher RTA temperatures.

**Fig. 5:**
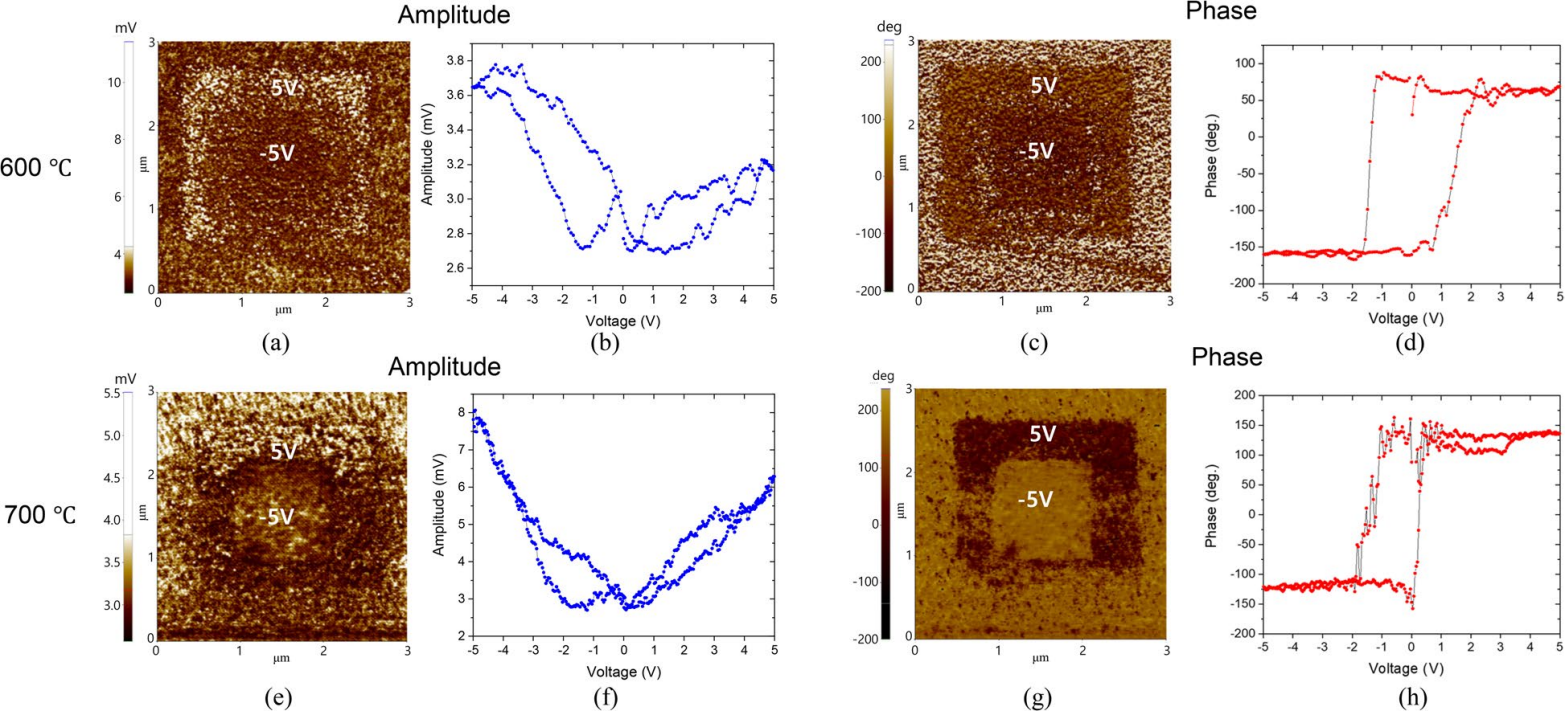
Piezoresponse force microscopy (PFM) amplitude and phase characterization of Hf_0.5_Zr_0.5_O_2_ thin films after rapid thermal annealing at 600 °C and 700 °C. **a**, **b** Amplitude image and corresponding amplitude–voltage hysteresis loop at 600 °C. **c**, **d** Phase image and corresponding phase–voltage hysteresis loop at 600 °C. **e**, **f** Amplitude image and amplitude–voltage hysteresis loop at 700 °C. **g**, **h** Phase image and phase–voltage hysteresis loop at 700 °C

Figure 5 presents the PFM amplitude and phase images, along with the corresponding hysteresis loops, for HZO films annealed at 600 °C and 700 °C. The amplitude signal reflects the electromechanical response, while the phase contrast and phase hysteresis loops provide direct evidence of polarization switching and ferroelectric domain formation.

For the film annealed at 600 °C, both amplitude and phase images show clear contrast between the + 5 V and − 5 V poled regions, indicating stable and well-defined ferroelectric domains. The corresponding hysteresis loops are sharp and well developed, demonstrating strong polarization switching and a robust electromechanical response. In contrast, the film annealed at 700 °C also exhibits domain switching, but with reduced amplitude and phase contrast, and less distinct hysteresis loops compared to the 600 °C sample. These features indicate a weakening of both the piezoelectric and ferroelectric responses.

Overall, the PFM results suggest that the ferroelectric behavior of HZO films is highly sensitive to annealing temperature. Annealing at 600 °C provides optimal conditions for the formation and stabilization of ferroelectric domains, whereas higher temperatures (e.g., 700 °C) likely modify the crystallinity or internal stress state, resulting in reduced domain stability and switching performance.

### Structural and electrical characteristics of TiN/HZO/TiN structures with various RTA temperatures

**Fig. 6:**
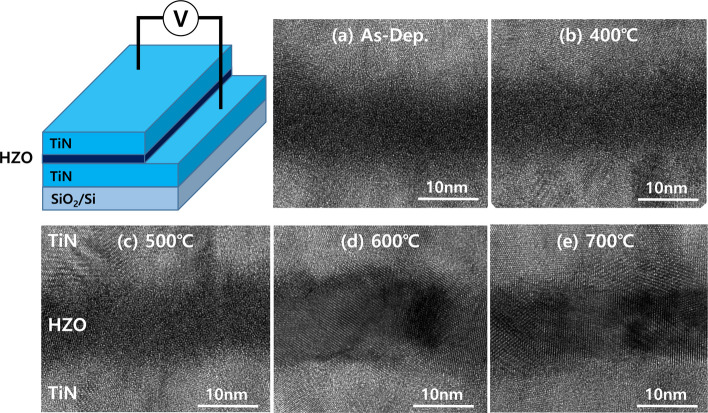
Cross‑sectional TEM images of TiN/HZO/TiN metal–insulator–metal capacitors after rapid thermal annealing (RTA) at different temperatures: (**a**) as‑deposited, (**b**) 400 °C, (**c**) 500 °C, (**d**) 600 °C, and (**e**) 700 °C. The left panel shows the measurement schematic and device stack used for electrical characterization, consisting of TiN/HZO/TiN on a SiO_2_/Si substrate in a top–bottom electrode configuration

To investigate the electrical properties of HZO ferroelectric thin films, metal–ferroelectric–metal (MFM) capacitor structures with TiN electrodes were fabricated, and their cross-sectional TEM images are presented in Fig. 6. TiN is widely used as an electrode material in HZO-based ferroelectric devices due to its compatibility and favorable electrical properties.

The TEM images of the TiN/HZO/TiN structures reveal a clear evolution of the HZO layer with increasing RTA temperature. In the as-deposited state, the HZO film appears amorphous, exhibiting no discernible lattice fringes. A similar amorphous structure is maintained at 400 °C, indicating insufficient thermal energy for crystallization. At 500 °C, the onset of crystallization becomes evident through the appearance of faint periodic lattice features. At 600 °C, the HZO layer shows well-defined lattice fringes and more developed grains, indicating a transition to a stable crystalline phase, likely the ferroelectric orthorhombic phase. At 700 °C, the film exhibits further enhanced crystallinity, with clearly defined grain boundaries and larger grain sizes compared to the 600 °C sample.

**Fig. 7:**
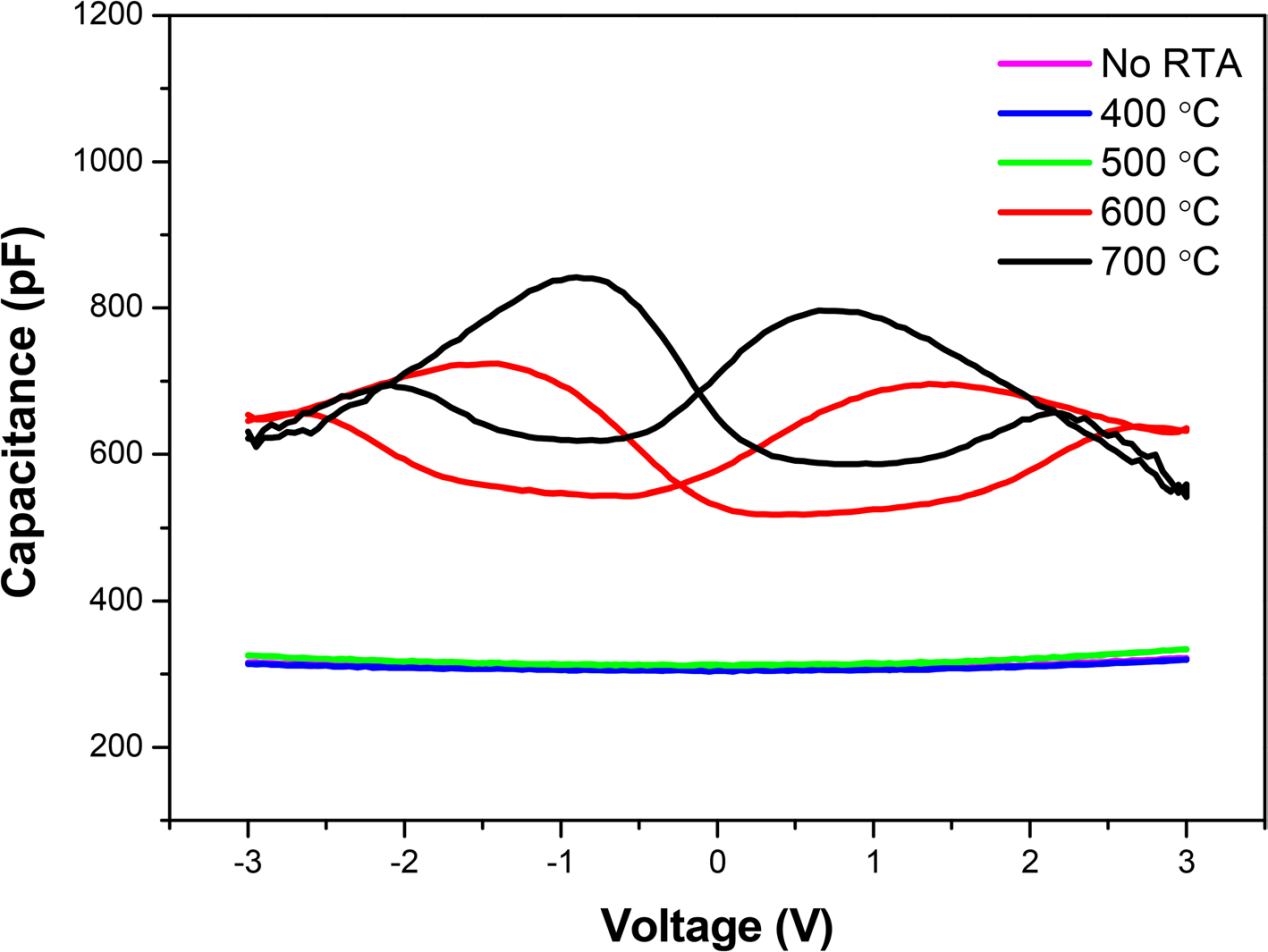
Capacitance–Voltage (C-V) curves of HZO films with various RTA temperature. Capacitance (C) as a function of applied voltage for HZO capacitors subjected to different rapid thermal anneal (RTA) conditions: no RTA, 400 °C, 500 °C, 600 °C, and 700 °C

To evaluate the ferroelectric switching behavior and dielectric properties of HZO thin films, capacitance–voltage (C-V) measurements were performed instead of conventional polarization–electric field (P-E) hysteresis loops. Although P-E measurements capture total polarization by integrating charges, they are easily distorted by leakage currents and non-ferroelectric effects, especially in ultra-thin films. In contrast, C-V analysis applies a small AC signal on top of a DC bias. This approach more accurately tracks voltage-dependent permittivity and clearly identifies true ferroelectric switching through the characteristic butterfly loop. Figure 7 shows the capacitance–voltage (C–V) characteristics of HZO films before and after rapid thermal annealing at temperatures between 400 °C and 700 °C. The as-deposited film and the samples annealed at 400 °C and 500 °C exhibit nearly constant capacitance values of approximately 310–320 pF over the entire voltage range. The absence of hysteresis or nonlinearity indicates that these films behave as linear dielectrics and do not exhibit ferroelectric switching.

In contrast, the films annealed at 600 °C and 700 °C display pronounced butterfly-shaped C–V curves with clear hysteresis, which is a hallmark of ferroelectric behavior arising from polarization reversal under an applied electric field. As the applied voltage approaches the coercive voltage, the capacitance increases, reaches a maximum, and then decreases after polarization switching. Among the annealed samples, the film treated at 700 °C exhibits the largest capacitance variation, with peak values approaching 840 pF, while the 600 °C sample shows a slightly lower maximum capacitance of approximately 720 pF but retains a well-defined butterfly loop. These results suggest that annealing above 600 °C promotes the formation of the ferroelectric phase in HZO films.

As shown in Fig. 5, the 600 °C sample exhibits a stronger local piezoresponse in the PFM measurements, which suggests a more pronounced local ferroelectric behavior and possibly a larger fraction or better uniformity of the ferroelectric orthorhombic phase in HZO (Kalinin et al. 2010; Park et al. 2015). In contrast, Fig. 7 shows that the 700 °C sample exhibits a larger capacitance modulation in the C–V measurements than the 600 °C sample, suggesting a stronger macroscopic dielectric response. Because C–V measurements can also be influenced by domain-wall motion, charge trapping, leakage, and non-ferroelectric phases, this behavior does not necessarily imply a larger remanent polarization (Damjanovic 1998; Pešić et al. 2016). Therefore, the PFM and C–V results are not contradictory but rather examine different aspects of the film’s electrical response.

## Conclusions

In this study, the influence of rapid thermal annealing (RTA) temperature on the crystallization and ferroelectric properties of HZO thin films was systematically investigated. Structural analyses using GI-XRD, TEM, and HAADF-STEM show that the films remain amorphous up to 500 °C and begin to crystallize near 600 °C, where the ferroelectric orthorhombic phase is most prominent. At higher temperatures, the non-ferroelectric monoclinic phase becomes increasingly dominant.

Electrical measurements reveal a clear correlation with this phase evolution. EFM indicates a gradual decrease in electrostatic response with increasing annealing temperature, while PFM confirms that the 600 °C sample exhibits the strongest domain contrast, polarization switching, and electromechanical response. The 700 °C sample retains ferroelectricity but with reduced domain stability and switching behavior.

C–V measurements of TiN/HZO/TiN capacitors further support these results: films annealed below 600 °C behave as linear dielectrics, whereas those annealed at 600 °C and 700 °C show characteristic ferroelectric hysteresis. Overall, an annealing temperature around 600 °C provides the optimal condition for stabilizing the orthorhombic phase and achieving enhanced ferroelectric performance.

## Data Availability

Data and materials will be made available upon request.

## Abbreviations

ALD: Atomic layer deposition
RTA: Rapid thermal annealing
PVD: Physical vapor deposition
TEM: Transmission electron microscopy
HRTEM: High-resolution transmission electron microscopy
HAADF-STEM: High-angle annular dark-field scanning transmission electron microscopy
GIXRD: Grazing incidence X-ray diffraction
EFM: Electrostatic force microscopy
PFM: Piezoresponse force microscopy

## References

[CR1] T.S. Böscke, J. Müller, D. Bräuhaus, U. Schröder, U. Böttger, Ferroelectricity in hafnium oxide thin films. Appl. Phys. Lett. **99**, 102903 (2011)

[CR2] A. Chernikova, M. Kozodaev, A. Markeev, D. Negrov, M. Spiridonov, S. Zarubin, O. Bak, P. Buragohain, H. Lu, E. Suvorova, A. Gruverman, A. Zenkevich, Ultrathin Hf_0.5_Zr_0.5_O_2_ ferroelectric films on Si. ACS Appl. Mater. Interfaces **8**, 7232–7237 (2016) 26931409 10.1021/acsami.5b11653

[CR3] D. Damjanovic, Ferroelectric, dielectric and piezoelectric properties of ferroelectric thin films and ceramics. Rep. Prog. Phys. **61**, 1267–1324 (1998)

[CR4] M.B. Hachemi, B. Salem, V. Consonni, H. Roussel, A. Garraud, G. Lefevre, S. Labau, S. Basrour, A. Bsiesy, Study of structural and electrical properties of ferroelectric HZO films obtained by single-target sputtering. AIP Adv. **11**(8), 2 (2021)

[CR5] T.D. Huan, V. Sharma, G.A. Rossetti, R. Ramprasad, Pathways towards ferroelectricity in hafnia. Phys. Rev. B **90**, 064111 (2014)

[CR6] S.V. Kalinin, A.N. Morozovska, L.Q. Chen, B.J. Rodriguez, Local polarization dynamics in ferroelectric materials. Rep. Prog. Phys. **73**, 056502 (2010)

[CR7] H.G. Kim, D.H. Hong, J.H. Yoo, H.C. Lee, Effect of process temperature on density and electrical characteristics of Hf_0.5_Zr_0.5_O_2_ thin films prepared by plasma-enhanced atomic layer deposition. Nanomaterials **12**(3), 548 (2022) 35159892 10.3390/nano12030548 PMC8839501

[CR8] R. Materlik, C. Künneth, A. Kersch, The origin of ferroelectricity in Hf_1−x_Zr_x_O_2_: a computational investigation and a surface energy model. J. Appl. Phys. **117**, 134109 (2015)

[CR9] T. Mikolajick, U. Schroeder, M.H. Park, Special topic on ferroelectricity in hafnium oxide: materials and devices. Appl. Phys. Lett. **118**, 180402 (2021)

[CR10] J. Müller, T.S. Böscke, D. Bräuhaus, U. Schröder, U. Böttger, J. Sundqvist, P. Kücher, T. Mikolajick, L. Frey, Ferroelectric Zr_0.5_Hf_0.5_O_2_ thin films for nonvolatile memory applications. Appl. Phys. Lett. **99**, 112901 (2011)

[CR11] J. Müller, T.S. Böscke, U. Schroder, S. Mueller, D. Brauhaus, U. Bottger, L. Frey, T. Mikolajick, Ferroelectricity in simple binary ZrO_2_ and HfO_2_. Nano Lett. **12**, 4318–4323 (2012) 22812909 10.1021/nl302049k

[CR12] M.H. Park, Y.H. Lee, H.J. Kim, Y.J. Kim, T. Moon, K.D. Kim, J. Mueller, A. Kersch, U. Schroeder, T. Mikolajick, C.S. Hwang, Ferroelectricity and antiferroelectricity of doped thin HfO_2_-based films. Adv. Mater. **27**, 1811–1831 (2015) 25677113 10.1002/adma.201404531

[CR13] M.H. Park, Y.H. Lee, H.J. Kim, T. Schenk, U. Schroeder, T. Mikolajick, C.S. Hwang, Review and perspective on ferroelectric HfO_2_-based thin films for memory applications. MRS Commun. **8**, 795–808 (2018)

[CR14] M. Pešić, F.P.G. Fengler, L. Larcher, A. Padovani, T. Schenk, E.D. Grimley, X. Sang, J.M. LeBeau, S. Slesazeck, U. Schroeder, T. Mikolajick, Physical mechanisms behind the field‐cycling behavior of HfO_2_‐based ferroelectric capacitors. Adv. Funct. Mater. **26**, 4601–4612 (2016)

[CR15] P. Polakowski, J. Müller, Ferroelectricity in undoped hafnium oxide. Appl. Phys. Lett. **106**, 232905 (2015)

[CR16] J. Robertson, High dielectric constant oxides. Eur. Phys. J. Appl. Phys. **28**, 265–291 (2004)

[CR17] X. Sang, E.D. Grimley, T. Schenk, U. Schroeder, J.M. LeBeau, On the structural origins of ferroelectricity in HfO_2_ thin films. Appl. Phys. Lett. **106**, 162905 (2015)

[CR18] S. Starschich, D. Griesche, T. Schneller, R. Waser, U. Böttger, Chemical solution deposition of ferroelectric yttrium-doped hafnium oxide films on platinum electrodes. Appl. Phys. Lett. **104**, 202903 (2014)

[CR19] S. Starschich, S. Menzel, U. Böttger, Pulse wake-up and breakdown investigation of ferroelectric yttrium doped HfO_2_. J. Appl. Phys. **121**, 154102 (2017)

[CR20] G.D. Wilk, R.M. Wallace, J.M. Anthony, High-κ gate dielectrics: current status and materials properties considerations. J. Appl. Phys. **89**, 5243–5275 (2001)

[CR21] G.T. Yu, G.H. Park, E.B. Lee, M.H. Park, Review of the mechanism for ferroelectric phase formation in fluorite-structure oxide. New Phys Sae Mulli **71**, 890–900 (2021)

